# Successful surgical treatment for intrahepatic arterioportal fistula with severe portal hypertension: a case report

**DOI:** 10.1186/s40792-019-0623-8

**Published:** 2019-04-23

**Authors:** Hideyuki Takata, Hiroshi Makino, Tadashi Yokoyama, Hiroshi Maruyama, Atsushi Hirakata, Junji Ueda, Hiroshi Yoshida

**Affiliations:** 1 Department of Surgery, Nippon Medical School Tama Nagayama Hospital, 1-7-1 Nagayama, Tama city, Tokyo 206-8512 Japan; 2 Department of Gastrointestinal Hepato-Biliary-Pancreatic Surgery, Nippon Medical School, 1-1-5 Sendagi, Bunkyo-ku, Tokyo, 113-8603 Japan

**Keywords:** Arterioportal fistula, Portal hypertension, Hepatectomy

## Abstract

**Background:**

Intrahepatic arterioportal fistula (IAPF) is a rare cause of portal hypertension. Interventional radiology (IVR) is generally selected as the first-line therapeutic option. Surgical treatment for IAPF is required in refractory cases of IVR. As the treatment success rate with IVR is high, cases requiring surgical treatment are extremely rare.

**Case presentation:**

A 54-year-old man was admitted to another hospital complaining of hematemesis due to rupture of the esophageal varices. A computed tomography revealed ascites and arterioportal fistula in the left lobe of the liver. Transcatheter arterial embolization (TAE) was performed to occlude the fistula; however, it could not reach complete occlusion. Thereafter, there were a total of four hematemeses, and six endoscopic variceal ligations were required. The second TAE also failed to reach complete occlusion. He was transferred to our hospital for further treatment. Because liver function was low due to frequent hematemeses and there was also uncontrollable ascites, it was confirmed that hepatectomy could not be performed safely at this time. Therefore, we ligated the left portal branch and ligated and dissected the left gastric vein to decrease portal vein pressure. However, on the 5th day after surgery, the esophageal varices reruptured. As the disappearance of ascites was observed in the postoperative course and the general condition also improved, left hepatectomy was performed to remove IAPF. There was no recurrence of portal hypertension for 1 year and 3 months since hepatectomy.

**Conclusions:**

This case was difficult to treat with IVR and required surgical treatment. Our experience in the present case suggests that hepatectomy to remove arterioportal fistula was considered effective for improving portal hypertension due to IAPF. However, careful treatment selection according to the patient’s overall condition and clinical course is necessary for IAPF presenting with severe portal hypertension.

## Background

Intrahepatic arterioportal fistula (IAPF) is defined as an abnormal communication between the hepatic artery and portal vein and might lead to severe portal hypertension [1]. IAPF, which induces portal hypertension, requires treatment. With recent developments in interventional radiology (IVR) techniques, IVR is the first-line treatment for IAPF [2]. Surgical treatment is required in refractory cases of IVR. However, in those requiring surgical treatment, there are cases with poor condition due to frequent hematemesis and/or poor control of ascites; therefore, careful selection of the surgical procedure is necessary. In the present study, we describe the case of a patient with IAPF who required left hepatectomy after ligation of the left portal vein and ligation and dissection of the left gastric vein.

## Case presentation

**Fig. 1:**
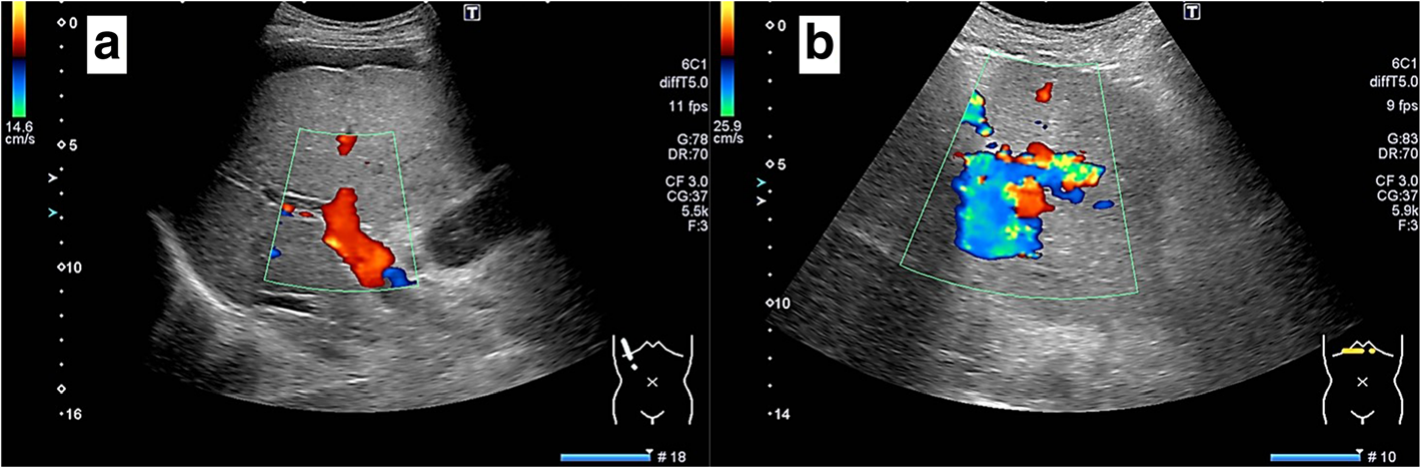
Color Doppler ultrasonography findings. **a** Hepatopetal flow was shown in the right branch of the portal vein. **b** Hepatofugal flow was shown in the left branch of the portal vein, and disturbed flow was shown around the left branch of the vein

**Fig. 2:**
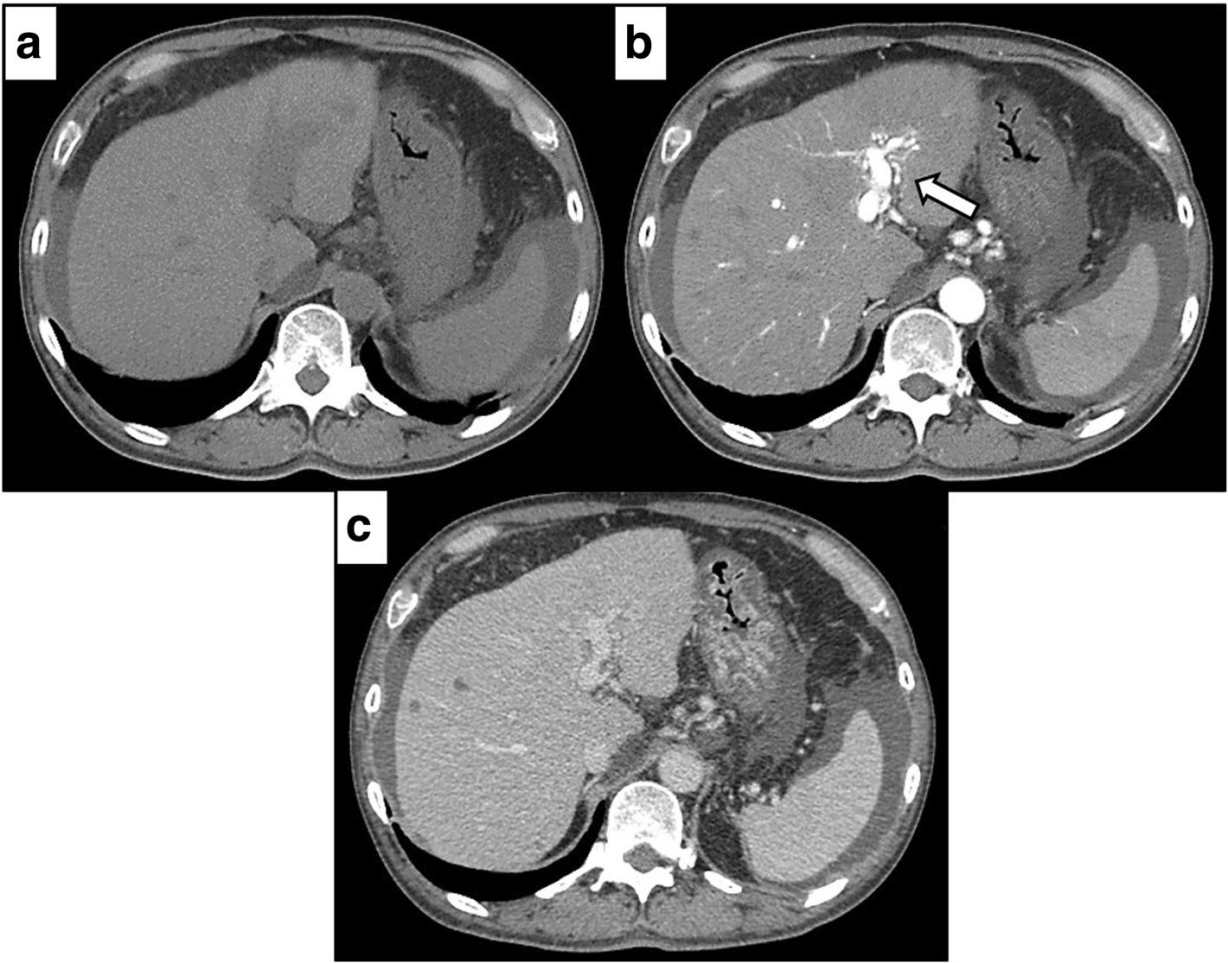
Contrast-enhanced computed tomography of the abdomen revealed ascites. Earlier enhancement of the umbilical part of the left branch of the portal vein in the hepatic arterial phase (arrow). **a** Plain. **b** Arterial phase. **c** Delayed phase

**Fig. 3:**
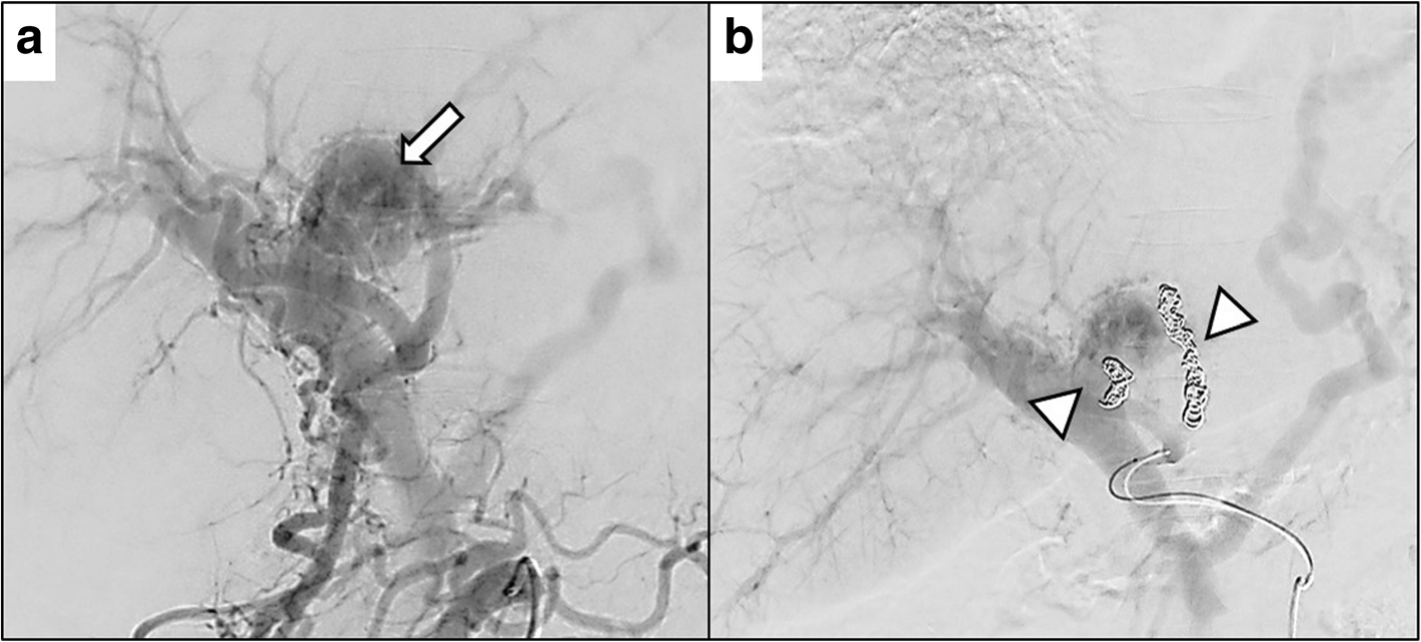
Digital subtraction angiography findings. **a** Superior mesenteric arteriography reveals intrahepatic arterioportal fistula (arrow) and early filling of the left branch of the portal vein. **b** Proper hepatic angiography after coil embolization of the A2, A3, and A4 (arrowhead) also shows incomplete occlusion of the fistula

**Fig. 4:**
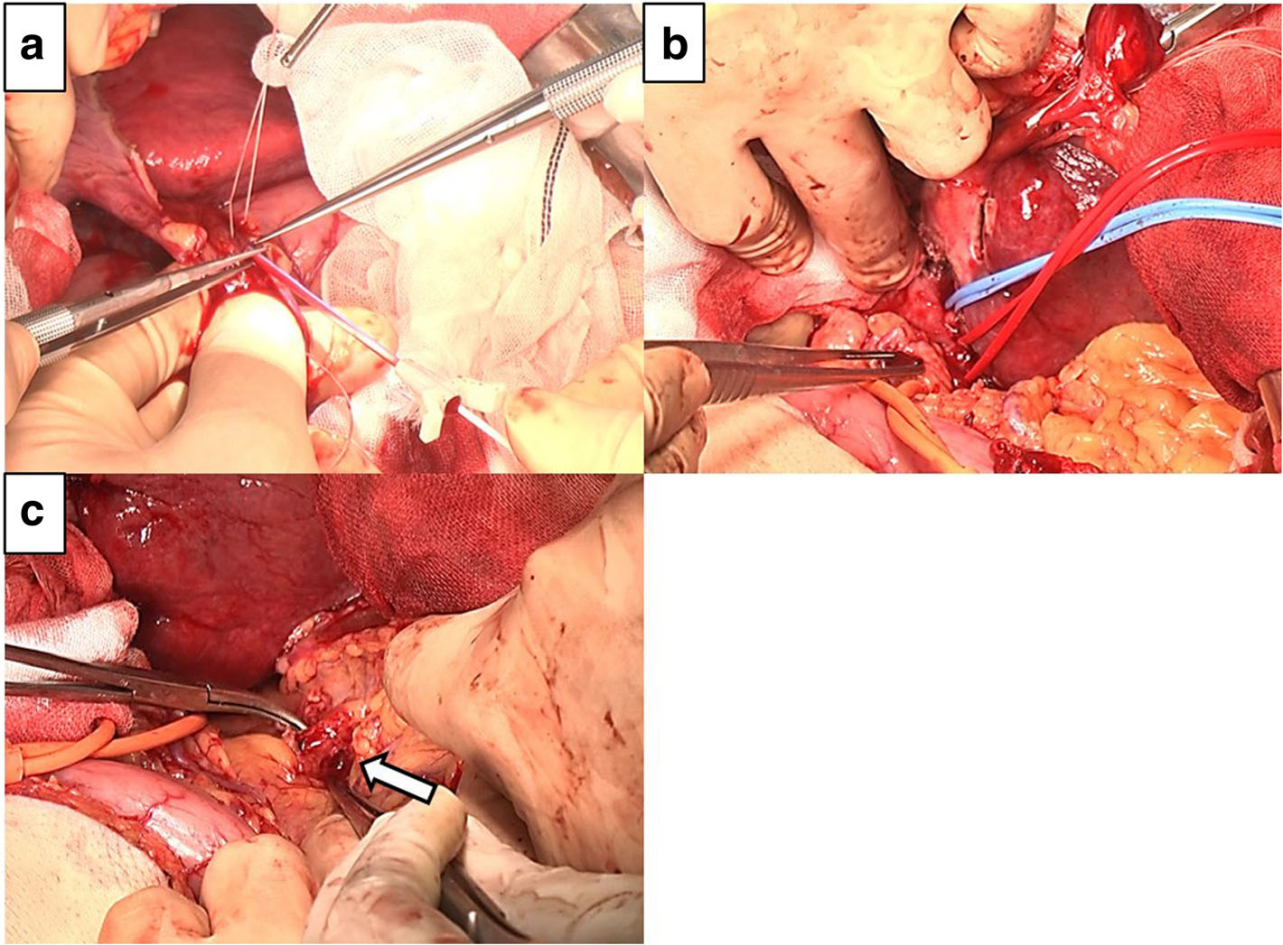
Intraoperative findings of the primary surgery. **a** A catheter was inserted from the paraumbilical vein, and the portal venous pressure was measured. **b** Identification of the left hepatic artery and left branch of the portal vein. **c** Identification of the left gastric vein (arrow), which supplies the varicose veins

**Fig. 5:**
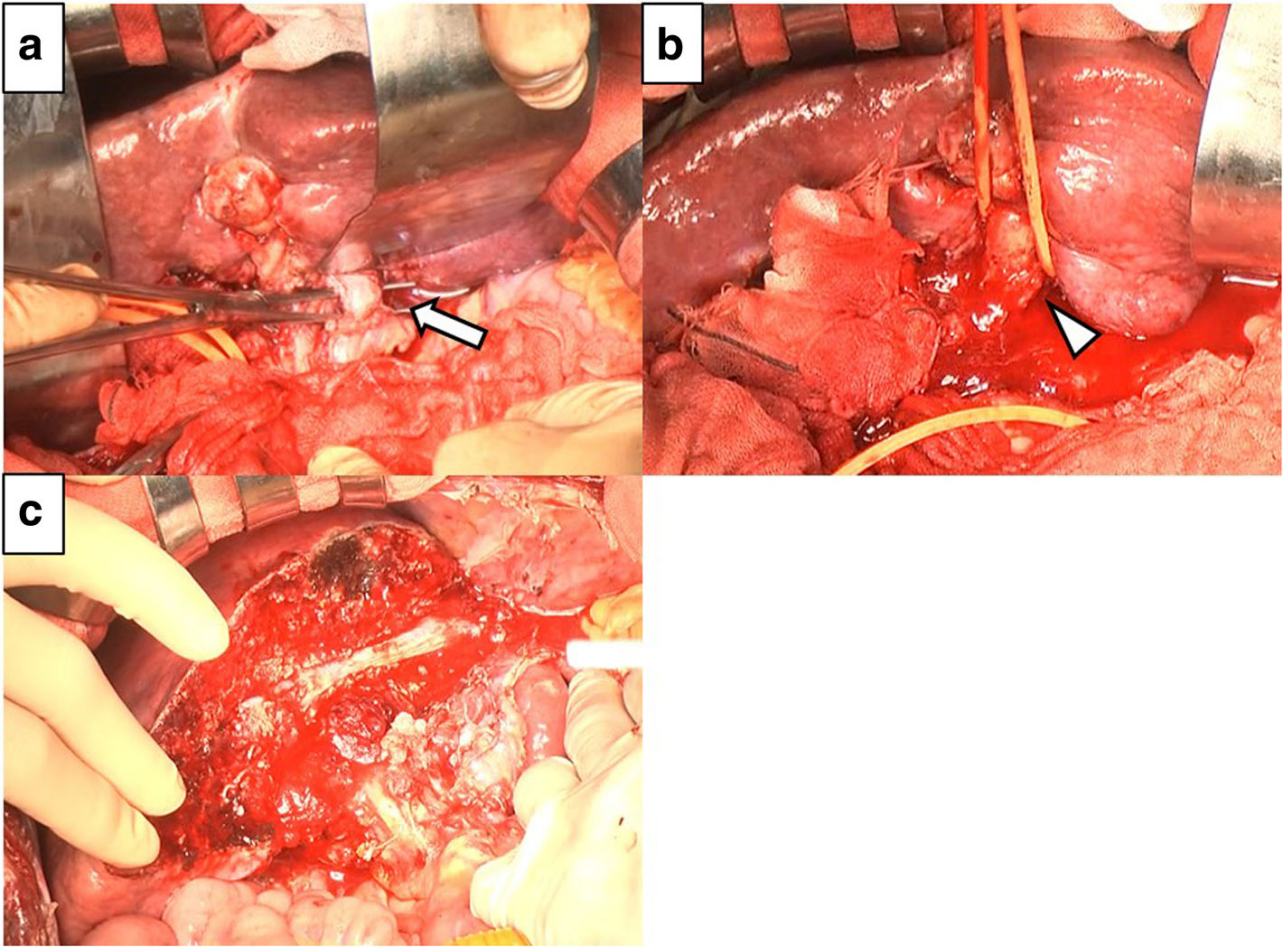
Intraoperative findings of the second surgery. **a** Identification of the left hepatic artery (arrow). Adhesion was observed because of the first surgery. **b** The left branch of the portal vein (arrowhead), firmly adhered. The left branch of the portal vein and left branch of the bile duct are taped together. **c** The main trunk of the middle hepatic vein is exposed in the transection plane

**Fig. 6:**
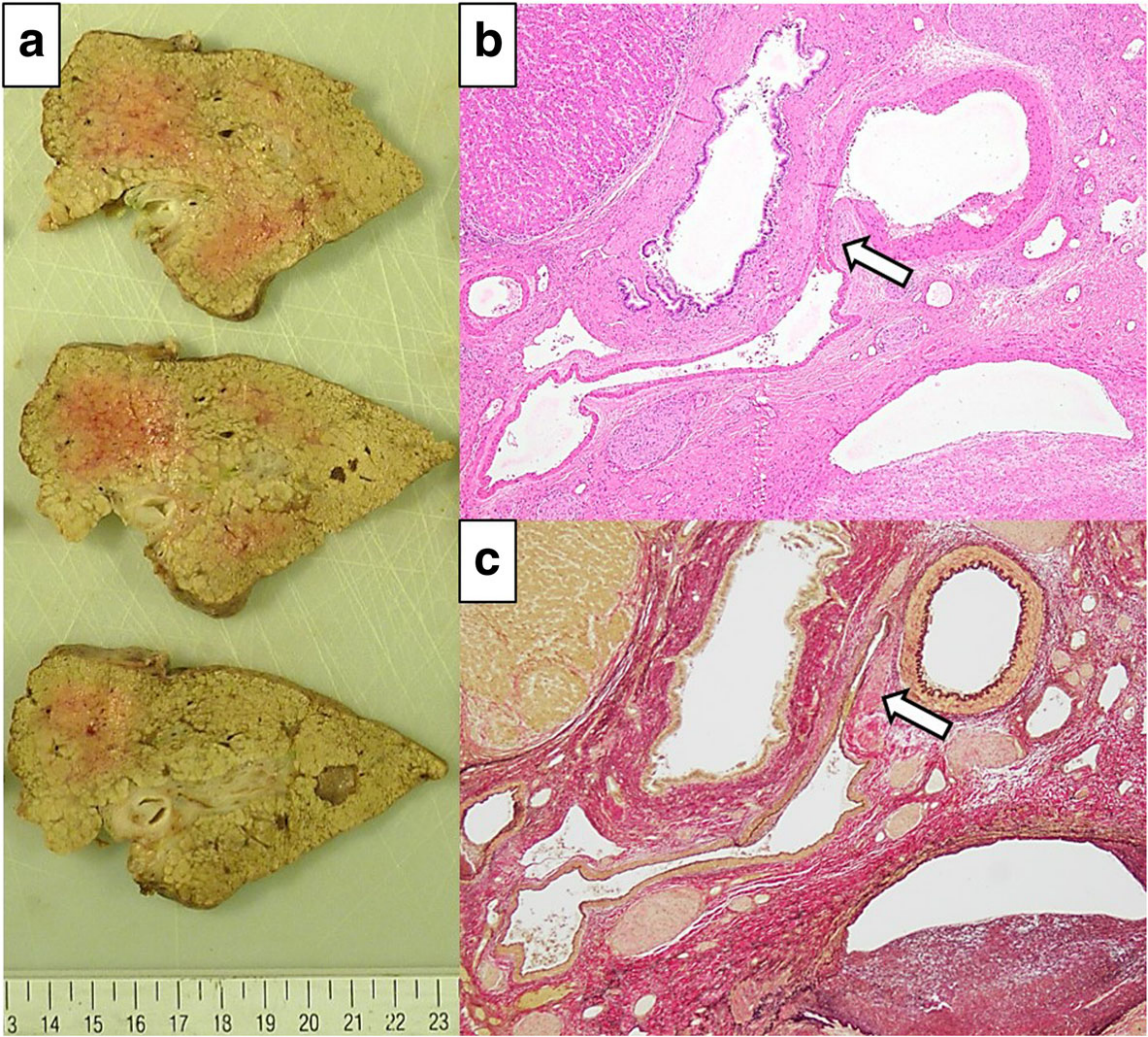
Macroscopic and microscopic findings. **a** Arterioportal fistula could not be identified macroscopically. **b** Many dilatated vessels can be observed in Glisson’s sheath. Arterioportal fistula (arrow) can also be observed (hematoxylin-eosin stain). **c** The portal vein and artery are distinguished with special staining. The same specimen shows an arterioportal fistula (Elastica van Gieson staining)

**Fig. 7:**
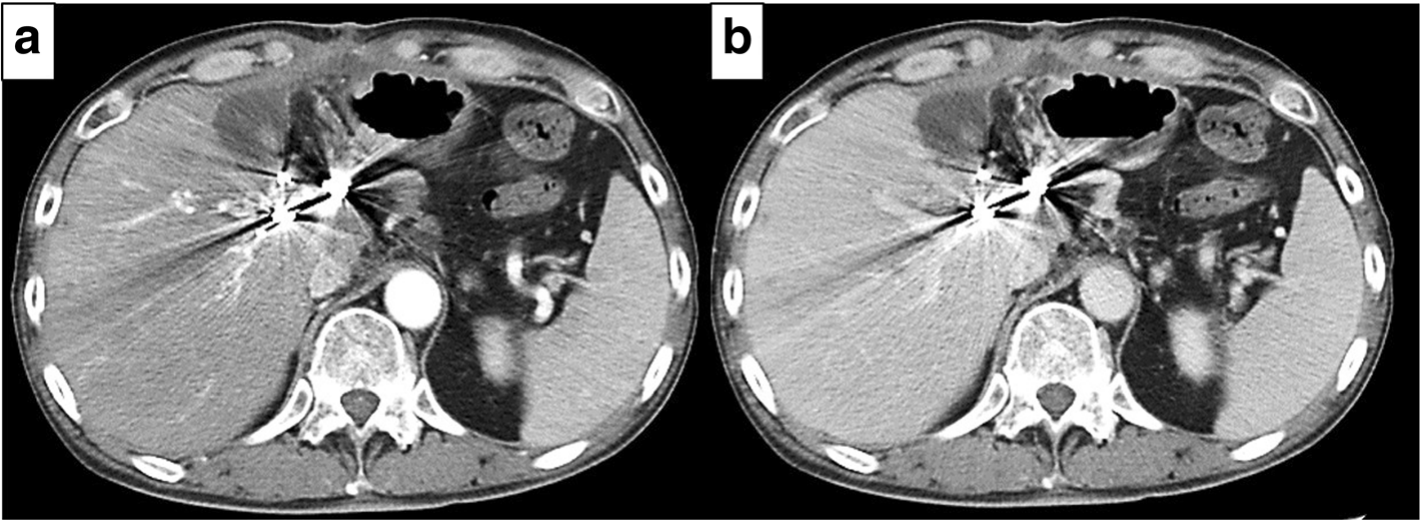
Contrast-enhanced computed tomography image of the abdomen after left hepatectomy. The earlier enhancement of the portal vein disappeared in the hepatic arterial phase. **a** Arterial phase. **b** Delayed phase

A 54-year-old man with type C cirrhosis was admitted to another hospital complaining of hematemesis due to rupture of the esophageal varices and underwent hemostasis with endoscopic variceal ligation (EVL). Abdominal ultrasonography revealed ascites, and color Doppler ultrasonography showed IAPF between the branch of the left hepatic artery and umbilical part of the left branch of the portal vein. The right portal venous flow was hepatopetal, and the left portal venous flow was hepatofugal (Fig. 1). Contrast-enhanced computed tomography (CT) demonstrated IAPF in the left lobe, and the umbilical part of the left branch of the portal vein was enhanced simultaneously in the arterial phase (Fig. 2). Digital subtraction angiography (DSA) revealed diffuse IAPF and an early filling of the left branch of the portal vein (Fig. 3a). The cause of portal hypertension was IAPF supplied by A2, A3, and A4, and transcatheter arterial embolization (TAE) using microcoils was performed to close the fistula. A2, A3, and A4 were embolized; however, the fistula was not completely occluded (Fig. 3b). Thereafter, there were a total of four hematemeses due to esophageal variceal rupture, and a total of six EVLs were performed. The second TAE also failed to reach complete occlusion because of diffuse collateralization. As hematemesis was repeated after treatment, the patient was transferred to our hospital for further treatment. Laboratory results were as follows: white blood cell count of 4500/μL (normal, 4000–9000); red blood cell count of 328 × 10^4^/μL (normal, 427–570 × 10^4^/μL); serum hemoglobin concentration of 10.2 g/dL (normal, 14–18 g/dL); serum platelet count of 12.8 × 10^4^/μL (normal, 15–35 × 10^4^/μL); aspartate transaminase concentration of 69 IU/L (normal, 8–38 IU/L); alanine transaminase concentration of 45 IU/L (normal, 4–44 IU/L); serum albumin concentration of 4.1 g/dL (normal, 3.9–4.9 g/dL); total bilirubin concentration of 0.6 mg/dL (normal, 0.2–1.2 mg/dL); prothrombin time of 67.0%; and ICGR15 level of 12.4%. The clinical Child-Pugh classification status was B. As with previous hospital examinations, abdominal CT demonstrated ascites and remaining IAPF in the left lobe of the liver. Although left hepatectomy including IAPF was thought to be needed, we concluded that major hepatectomy at this point had a high risk because of poor general condition due to frequent hematemesis and deterioration of liver function. Although an apparent decline in liver function due to frequent massive bleeding was possible, the general condition was extremely poor and was not suitable for left hepatectomy. Therefore, we performed ligation of the draining left portal vein and dissection of the left gastric vein that supplied varicose veins (Fig. 4). A catheter was inserted from the paraumbilical vein to measure the portal venous pressure. Portal venous pressure decreased from 330 to 210 mmH_2_O after ligation of the left portal vein. The operating time was 251 min, and the intraoperative bleeding was 340 mL. However, melena appeared on the 5th postoperative day, and the progression of anemia was observed. An emergency upper gastrointestinal endoscopy was performed on suspecting bleeding from the esophageal varices. Although there was no active bleeding, EVL was performed for the esophageal varices with red color signs. The laboratory results on the 14th postoperative day were as follows: aspartate transaminase concentration, 48 IU/L; alanine transaminase concentration, 34 IU/L; serum albumin concentration, 3.6 g/dL; total bilirubin concentration, 0.5 mg/dL; prothrombin time, 67.9%; and ICGR15 level, 13.8%. Ascites disappeared at the CT findings in the postoperative course, and the clinical Child-Pugh classification status improved from grade B to grade A. After the first surgery, the general condition and liver function were improved on the 14th postoperative day. Therefore, left hepatectomy (Fig. 5) was performed to remove the IAPF completely on the 21st postoperative day. Adhesion was observed around the hepatic hilum because of the first operation. Furthermore, the division of the hepatic hilum was hemorrhagic owing to portal hypertension. As the left portal vein was ligated at the time of the first operation, the demarcation line was found on the liver surface by dissection of the left hepatic artery. After mobilization of the left liver, parenchymal dissection was performed under intermittent inflow occlusion, that is, 15 min of occlusion followed by 5 min perfusion. The operating time was 318 min, and the intraoperative bleeding amount was 1800 mL. In the macroscopic findings of the resected specimen, arterioportal fistula could not be identified (Fig. 6a). In the microscopic findings, the background liver tissue showed the presence of many pseudo-nodules, indicating liver cirrhosis. Many dilatated vessels in Glisson’s sheath and arterioportal fistula were observed (Fig. 6b). Contrast-enhanced CT after left hepatectomy revealed that earlier enhancement of the branch of the portal vein disappeared in the hepatic arterial phase (Fig. 7). Although anorexia and wound infection were noted, there were no other major complications, and he was discharged on the 32nd postoperative day. There was no recurrence of portal hypertension for 1 year and 3 months after hepatectomy.

## Discussion

IAPF is defined as an intrahepatic communication between the hepatic artery and portal venous system, which can be an uncommon cause of presinusoidal portal hypertension [1, 3]. The cause is classified as congenital or acquired. The congenital factor is extremely rare and acquired factors account for the majority of cases [1]. Percutaneous liver biopsy [4], percutaneous transhepatic biliary drainage [5], and blunt or penetrating liver trauma [6, 7] may injure the hepatic artery and portal vein and lead to the development of IAPF. Hepatocellular carcinomas [8], cirrhosis [9], and cavernous hemangiomas [10] can also result in IAPF. In this case, it was speculated that type C cirrhosis has some influence on IAPF formation because there was no history of trauma, liver biopsy, and liver tumor.

Small asymptomatic cases of IAPF can be followed up and do not require treatment. In contrast, IAPF with portal hypertension as in our case and an increasing trend requires treatment [11]. The therapeutic approaches for IAPF are IVR and surgery. Previously, the first-line treatment was ligation of the hepatic artery supplying the IAPF or simple resection of IAPF [12]. Recently, IVR is considered the first-choice treatment in place of surgery due to the slightly high operative mortality and rapid development of IVR techniques [13, 14]. IVR has a success rate of over 90% for IAPF cases, and a few cases requires surgical treatment [15]. Diffuse and intrahepatic IAPF may be difficult to treat with IVR, and surgical treatment should be considered. In the present case, TAE was performed twice and failed to reach complete occlusion of the IAPF. As a next-line treatment, trans-ileocecal portal vein embolization [16] was considered to reduce portal vein pressure. However, the portal pressure was predicted to be high owing to the anastomosis between the artery and portal vein; therefore, we could not deny the possibility that the embolic agent could be swept away and embolize the right portal branch when releasing the balloon. A similar procedure is balloon-occluded retrograde transvenous obliteration [17], and long-term balloon placement is required to avoid the flow of the embolic agent. We considered that balloon placement for long-term retention is difficult to attain with laparotomy. Therefore, we decided to choose surgical treatment.

In previous reports, ligation of the hepatic artery [18], hepatectomy [19], and liver transplantation [20] were performed in refractory cases of IVR. The operative procedure should be selected according to the patient’s general condition and hepatic reserve. Simple surgical ligation of feeding arterial branches had been used selectively. However, in cases with multiple arterial collateral branches to the IAPF, simple ligation alone can often be ineffective [21]. Considering poor general condition due to frequent hematemesis, poor control of ascites, and deterioration of the hepatic reserve, we concluded that hepatectomy was ineffective as a first-line treatment in the present case. Therefore, we performed ligation of the left portal vein with hepatofugal flow and dissection of the left gastric vein that supplied varicose veins in primary surgery. Although it seemed to be effective, rupturing of the esophageal varices occurred after surgery, which was insufficient as a treatment for portal hypertension in the present case. Since the general condition did not worsen from primary surgery and ascites also disappeared, it was confirmed that hepatectomy was possible.

The long-term outcome of hepatectomy for refractory IAPF cases of IVR is relatively good, and recurrence of portal hypertension is not observed for a long period including that in our case [12, 19]. For refractory IAPF cases of IVR, hepatectomy is an effective therapeutic tool when the liver reserve is maintained.

## Conclusions

We encountered a case of IAPF with portal hypertension that was difficult to treat with IVR because of diffuse fistulas. TAE is the first-line treatment for all IAPF cases with severe portal hypertension. Surgical treatment should be chosen for cases refractory to TAE. In IAPF cases, portal pressure is predicted to be high owing to the anastomosis between the artery and the portal vein. Embolizing the branch of the portal vein with the IVR technique to reduce the portal vein pressure may cause embolic material to be swept away, and we think that the risk is high in IAPF cases. Surgical treatment should be selected for refractory cases of TAE. Hepatectomy for IAPF seems to be effective from the viewpoint of long-term outcome. Although it may be difficult to decide which type of surgery is preferred in cases of poor general condition or liver reserve, it is necessary to select an adequate treatment according to the patient’s condition.

## Abbreviations

CT: Computed tomography
DSA: Digital subtraction angiography
EVL: Endoscopic variceal ligation
IAPF: Intrahepatic arterioportal fistula
IVR: Interventional radiology
MRI: Magnetic resonance imaging
TAE: Transcatheter arterial embolization
